# Application of Deep Convolution Network to Automated Image Segmentation of Chest CT for Patients With Tumor

**DOI:** 10.3389/fonc.2021.719398

**Published:** 2021-09-29

**Authors:** Hui Xie, Jian-Fang Zhang, Qing Li

**Affiliations:** ^1^ Department of Radiation Oncology, Affiliated Hospital (Clinical College) of Xiangnan University, Chenzhou, China; ^2^ Key Laboratory of Medical Imaging and Artifical Intelligence of Hunan Province, Chenzhou, China; ^3^ Department of Physical Examination, Beihu Centers for Disease Control and Prevention, Chenzhou, China; ^4^ School of Medical Imaging and Rehabilitation, Xiangnan University, Chenzhou, China

**Keywords:** deep learning, convolutional network, medical image segmentation, similarity coefficient, radiotherapy

## Abstract

**Objectives:**

To automate image delineation of tissues and organs in oncological radiotherapy by combining the deep learning methods of fully convolutional network (FCN) and atrous convolution (AC).

**Methods:**

A total of 120 sets of chest CT images of patients were selected, on which radiologists had outlined the structures of normal organs. Of these 120 sets of images, 70 sets (8,512 axial slice images) were used as the training set, 30 sets (5,525 axial slice images) as the validation set, and 20 sets (3,602 axial slice images) as the test set. We selected 5 published FCN models and 1 published Unet model, and then combined FCN with AC algorithms to generate 3 improved deep convolutional networks, namely, dilation fully convolutional networks (D-FCN). The images in the training set were used to fine-tune and train the above 8 networks, respectively. The images in the validation set were used to validate the 8 networks in terms of the automated identification and delineation of organs, in order to obtain the optimal segmentation model of each network. Finally, the images of the test set were used to test the optimal segmentation models, and thus we evaluated the capability of each model of image segmentation by comparing their Dice coefficients between automated and physician delineation.

**Results:**

After being fully tuned and trained with the images in the training set, all the networks in this study performed well in automated image segmentation. Among them, the improved D-FCN 4s network model yielded the best performance in automated segmentation in the testing experiment, with an global Dice of 87.11%, and a Dice of 87.11%, 97.22%, 97.16%, 89.92%, and 70.51% for left lung, right lung, pericardium, trachea, and esophagus, respectively.

**Conclusion:**

We proposed an improved D-FCN. Our results showed that this network model might effectively improve the accuracy of automated segmentation of the images in thoracic radiotherapy, and simultaneously perform automated segmentation of multiple targets.

## Introduction

As medical imaging technology and computer technology are being increasingly applied in the field of oncology radiotherapy, radiotherapy has now developed to a stage where precision radiotherapy, characterized by image-guided and adaptive radiotherapy, became predominant (1, 2). Precision radiotherapy requires precise delineation of the target area and organs at risk, accompanied by online image-guided therapeutic irradiation, as well as the modification and adjustment of subsequent radiotherapy plans, which ultimately aimed to ensure the delivery of the effective dose to the target while avoiding normal tissues and organs. In current practices of clinical radiation therapy planning, the delineation of the target area and organs at risk usually involves manual work of experienced radiologists and tumor radiotherapy physicists, which is a time- and labor-intensive process. The accuracy and efficiency rely heavily on the clinical experience of physicians and physicists, and it cannot avoid the large variability between delineators. The development of computer-automated processing and artificial intelligence is driving rapid advances in automated and semi-automated delineation algorithms based on various computational image processing techniques, some of which have been put into clinical practices, including segmentation algorithms based on features of image gray level, color and texture, nonlinear diffusion algorithms using level set model, automated segmentation algorithms based on templates, and machine learning algorithms based on manually extracted features (3). However, these semi-automated and automated segmentation algorithms are still immature. Especially when boundaries between organ tissues are not obvious, the performance of automated segmentation is particularly unsatisfactory. The template-based algorithm requires a lot of running time due to the compositions of the template library, while the recognition of image features depending on professional experience is not necessarily ideal. Besides, most of the current algorithms are designed for a single organ or tissue, thereby being incapable of auto-segmenting multiple organs or tissue, which results in the inefficiency of clinical work.

In recent years, artificial intelligence technologies based on deep learning have presented tremendous opportunities for various fields including clinical medicine. Deep convolutional neural network (DCNN), or convolutional neural network (CNN) (4), is widely used in computer image recognition and more and more in the research of automated segmentation of medical images. For example, the U-net DCNN proposed by Olaf et al. (5) was applied to biomedical image recognition to achieve automated segmentation of biological cell images. When the DCNN is applied to medical image segmentation, image features can be extracted layer by layer from low to high through multi-layer convolution operation, and the automatically extracted features are correctly classified through iterative training and learning of calibration datasets, so as to achieve simultaneous segmentation of multi-structure targets (6–8). If we combine the trained DCNN model and graphic processing units (GPU) hardware acceleration, computed tomography (CT) images of tissues or organs experiencing radiotherapy can be segmented rapidly, and the structure of the target area and organs at risk can be accurately delineated automatically, which will promote the further development of precision radiotherapy.

## Materials And Methods

### Patient Datasets and Computer Working Platform

For this experiment, we collected the image data from the image database of clinical radiotherapy cases established at the early phase by the Department of Radiotherapy of Affiliated Hospital of Xiangnan University. Our research team searched the image database according to the disease type and structure, with search items such as lung cancer, left lung, right lung, pericardium, trachea, and esophagus, and eventually obtained the image data of clinical lung cancer cases undergoing radiotherapy. The image data included chest CT scan sequences of desensitized patients and the corresponding files of organ structure contour. With the aid of relevant medical image processing technology that analyzed and extracted the contouring data of the structure of each organ in the images, the organ delineation atlas corresponding to each slice image on the CT image sequence was thus generated.

**Figure 1:**
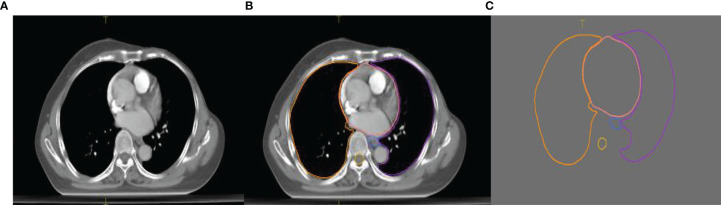
Images of a lung patient in the experimental data. **(A)** is the original axial slice image of CT scan; **(B)** is the axial slice image of CT scan delineated by the physician; **(C)** is the contour of delineated organs extracted after processing.

The experimental data set contained a total of 120 sets of chest CT images. Among them, 70 sets were randomly selected as the training set that included 8,512 axial slice images and organ delineation contour maps; 30 sets were randomly selected as the validation set that included 5,525 axial slice images and organ delineation atlases; 20 sets were randomly selected as the test set that included 3,602 axial slice images and organ delineation atlases. **Figure 1** is one of the examples, in which **Figure 1A** is the axial slice image of the patient and **Figure 1B** is the organ atlases delineated by the physician.

This study was performed on an ultramicro 4028GR-TR computer server. Its hardware system contained two Intel E5-2650V4 models of CPU, 128 GB of memory, 3 TB of SSD hard disk, and 8 GPUs of NVIDIA GeForce 1080Ti model; the software system included Ubuntu Server 16.04 operating system, CUDA8.0 and cuDNN6.0, and the latest Caffe deep learning framework.

### Optimization and Improvement of Fully Convolutional Network

The basic mechanism of the FCN proposed by Shelhamer et al. (9) is that FCN extracts image features through convolution, performs feature compression for feature image pooling processing, obtains segmented images as big as the original image through upsampling, and then optimizes output adjustment with the jump structure.

Six published networks were used in our study, including FCN based on the VGG16 (10) algorithm, the DeepLab series proposed by Chen et al., and the U-net (5, 9, 11, 12), as well as three dilation fully convolutional networks (D-FCN) modified by our research team through combining the deep learning methods of fully convolutional network and atrous convolution (AC). It’s reported that systematic dilation supports exponential expansion of the receptive field without loss of resolution or coverage, which increases the accuracy of state-of-the-art semantic segmentation systems (13). We used the training dataset for tuning and training to obtain the optimal network models for the forementioned chest image segmentation by comparing and comprehensively analyzing the automated segmentation results and manual delineation results of each network training model.

Preprocessing of data: Because the pre-training models selected for this study are based on the results of training with RGB three-channel natural images, and the medical image sets used in this study are single-channel CT images, it is necessary to construct the single-channel medical image into three-channel image in the data input layer. In the present study, we made two copies of the original image data to constitute virtual three-channel medical image data.

Published model training: We selected 5 DCNNs based on the FCN VGG16 algorithm, including FCN 32s, FCN 16s, FCN 8s, DeepLab-largeFOV, and DeepLabv2-VGG16, and 1 U-net model, which are suitable for image segmentation. We also leveraged these models trained on other natural image data sets as pre-trained models. We modified and optimized the pre-trained models, including changing the data input layer to adapt it to the data format of the medical image in our datasets. We added the window adjustment layer by combining the difference between medical images and natural images. In our study, the [-300, 600] window width was divided into three equal parts according to the characteristics of the window values of each structure of chest CT. The equally divided value range was the window width, and the median value was the window value. The window was adjusted for each channel separately. We set the number of characteristic maps of the output layer according to the category of the target that the experiment was designed to segment, and used the images in the training set to perform 500,000 repeated iterations for tuning and training these network models, so as to obtain the optimal training result of each network. In addition, it is necessary in the training process to adjust and optimize the training hyperparameters as actual training situations might change – specifically, learning strategy, initial learning rate, batch size, momentum, weight decay rate, etc., to improve the prediction accuracy of the model.

**Figure 2:**
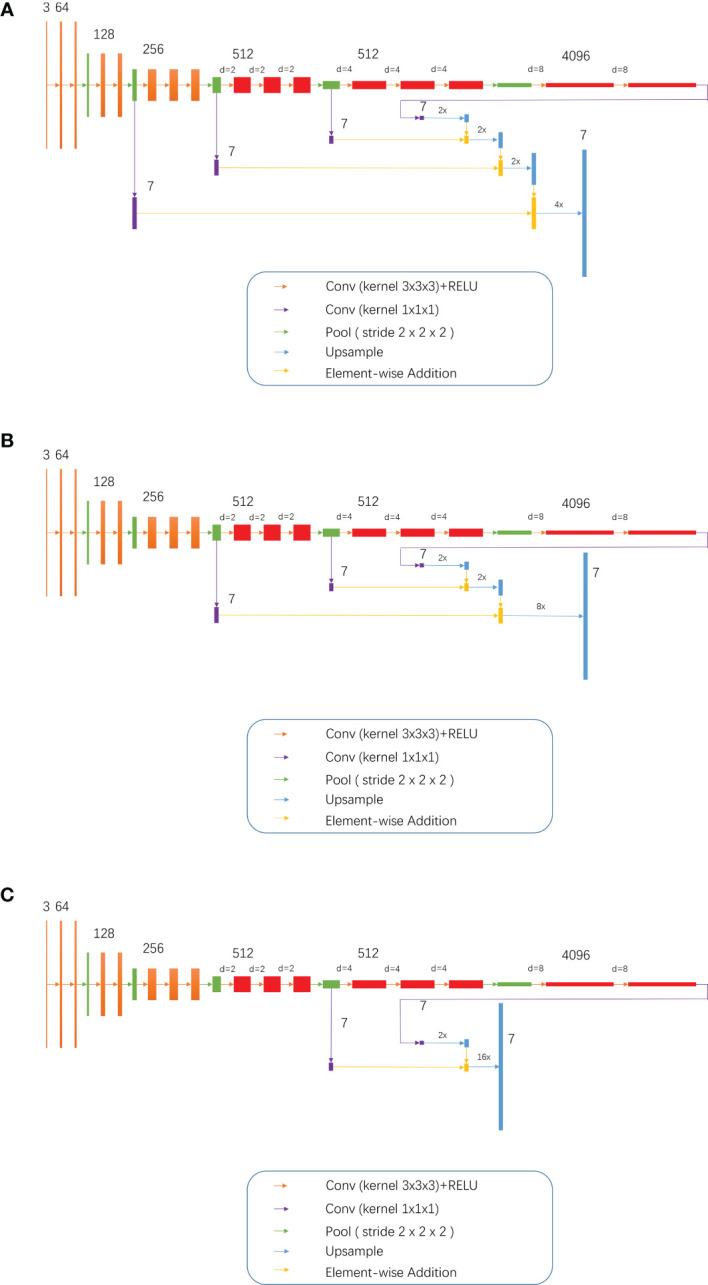
3, 7, 64, 128, 512 and 4096 meant 3 7, 64, 128, 512 and 4096 image channels, respectively; d meant d-1 dilation were plug in between every two elements of the convolution kernel; 2x and 4x are multiples of upsampling. **(A)** D-FCN 4S; **(B)** D-FCN 8S; **(C)** D-FCN 16S.

Training of the modified models: While combining the characteristics of FCN and the idea of atrous convolution, the pool3, pool4, and pool5 of the FCN 32s network, as well as part or all of the subsequent convolutional layers, were modified into dilation convolutional layer, namely, the so-called D-FCN. A total of 3 modified FCN models were thereby generated: D-FCN 4s, D-FCN 8s, and D-FCN 16s (**Figure 2**). Similarly, we employed the same datasets to tune and train the modified D-FCN models with the FCN32s network model as a pre-training model.

Optimal model validation: During the training process, a series of training models were generated with every 5,000 iterations as an observation mirror image. The manually delineated structural contour regions in the 5,525 images of the 30 patients in the validation set were used as the prediction targets to validate the segmentation consistency of the training models that were obtained from the training of the above 9 networks, respectively. We worked out the Dice coefficient by calculating the similarity between the automated segmentation results of the training models and the manual delineation results, and thus drew the Dice curve of the training models under different iterative mirrors of each network. Finally, we found the optimal segmentation model of each network by analyzing the Dice curve.

### Automated Image Segmentation Test of Network Models

The manually delineated contour regions of 20 sets of 3,602 axial slice images in the test set were used as the prediction targets. The optimal segmentation models selected above were employed to perform the automated segmentation of the targets so that we could test the effectiveness of each network model and the accuracy of automated segmentation. We calculated the similarity between the automated segmentation results and the manual delineation results in terms of global and individual organ structures, respectively. We compared the Dice coefficients and comprehensively evaluated each network model while considering the speed of automated segmentation processing.

### Evaluation Indicators

As we all know, intersection-Over-Union (IoU) and Dice coefficient are both important and common indicators for segmentation neural network assessment. The previous report which compared Dice coefficient with IoU, indicated that using Dice could have higher score than IoU (14). Therefore, in this paper, Dice coefficient is used to evaluate the effect of automated segmentation by network models, that is, to evaluate the similarity between the automated image segmentation results and the manual delineation results of physicians. Dice is calculated by:


$$\text{Dice}(\text{X},\text{Y})=\frac{2*\left|\text{X}\cap \text{Y}\right|}{\left|\text{X}\right|+\left|Y\right|}$$

Where X denotes the set of pixels for the automatically segmented image, Y denotes the set of pixels for the manually delineated image, | X ∩ Y | is the intersection of two sets of pixels, and | X + | Y | is the union set of the both. The range of Dice is [0, 1], and the higher the value of Dice is, the closer the result of automated segmentation is to that of manual delineation. In this paper, we calculated not only the global Dice of all segmented target regions, but also the Dice of individual segmented target region, so as to evaluate the effect of automated segmentation by the model more comprehensively.

## Results

In our study, the training set was comprised of 70 sets of 8,512 CT axial slice images of patients undergoing pulmonary radiotherapy, as well as organ atlases manually delineated by radiologists. Nine deep networks, including 6 published networks and 3 networks modified by us, were tuned and trained for automated image segmentation, respectively. 30 sets of 5,525 CT images, as well as manually delineated organ contour atlases, constituted the validation set, and were used to validate the consistency of the models obtained from tuning and training. The optimal segmentation model of each network was determined by Dice analysis. Finally, the effectiveness and accuracy of the optimal segmentation model of each network were tested by a test set containing 20 sets of 3,602 CT images, and the performance of each model in automated image segmentation of radiotherapy localization was comprehensively evaluated.

### Training and Optimization Results of Network Model

**Figure 3:**
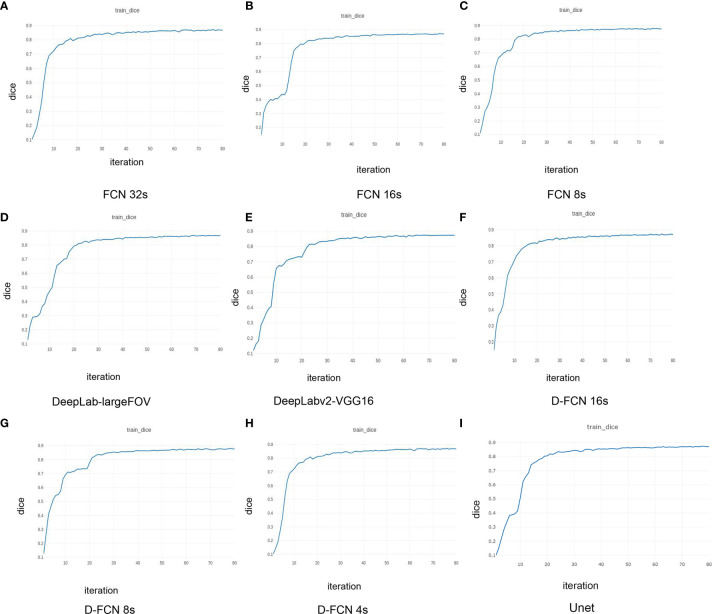
| Dice curves of the training effects of the 8 network models. **(A)** is the Dice curve of the training effect of FCN 32s network; **(B)** is of FCN 16s network; **(C)** is of FCN 8s network; **(D)** is of DeepLab-largeFOV network; **(E)** is of DeepLabv2-VGG16 network; **(F)** is of D-FCN 16s network; **(G)** is of D-FCN 8s network; **(H)** is of D-FCN 4s network; **(I)** is of Unet network.

**Figure 3** presents the Dice curves for the training and validation processes of the 9 networks. The Dice value of each network model increased with the number of iterations during the training. The convergence of Dice values showed that the improved D-FCN 4s model constructed in this study (**Figure 3H**) had the fastest convergence rate and the best stable convergence rate compared with the other models.

**Table 1** shows the statistical results of the iterative operation of automated segmentation of organs for each model, including the optimal Dice score and the number of iterations when reaching the optimal value. All the models in our study presented high global optimal Dice, which suggested that the automated segmentation results were close to the expert delineation results. The D-FCN 4s model proposed in this paper had the highest global Dice (87.11%) compared with the other models, indicating that it had superior performance in automated segmentation to the other models.

**Table 1:** Iterative operation results of automated organ segmentation for the 8 network models.

Network Model	Global Dice/%	95% CI		Best epoch (× 10000)
		Lower	Upper	
FCN 32s	86.32	78.25	93.50	77
FCN 16s	86.51	78.68	93.69	80
FCN 8s	86.95	79.23	93.80	78
Deeplab-largeFOV	86.36	77.94	93.67	76
Deeplabv2-VGG16	86.89	79.00	93.93	73
D-FCN 16s	86.47	78.09	93.82	72
D-FCN 8s	87.05	79.11	94.01	78
D-FCN 4s	87.11	79.40	93.95	78
Unet	86.81	78.96	93.75	74

CI, confidence interval.

### Test Results and Analysis of Automated Segmentation

**Table 2** shows the test results of automated segmentation of target structures for the 9 models by using the images in the test set. The table lists the global Dice of one-time automated segmentation of 6 target organs of each test case by different models, the optimal Dice of individual organ structure, and the automated segmentation operation time of each model. The comparison between the automated segmentation results of each model for the test set or the validation set both showed that D-FCN4s has a better segmentation effect than or is equivalent to the other network models, regardless of the global Dice or the Dice of the individual structure. Regarding automated segmentation operation time, D-FCN4s was slower in prediction segmentation than the other models because it preserved more image details for the sake of a finer segmentation effect. There was no downsampling operation above the Pool3 layer, and as a result, the resolution of the feature image in the following layers was larger, so the amount of computation increased greatly and the speed of prediction became slower. However, the predicted automated delineation speed of DFCN4s, which took less than 3 minutes on average, was acceptable in the practice of radiotherapy

**Table 2:** Test results of the optimal segmentation models of the 8 network models.

Network Model	Dice/%							Average time/s
	Global	Lung (L)	Lung (R)	Heart	Esophageal	Trachea	Spinal Cord	
FCN 32s	86.32	97.15	96.75	88.87	69.13	84.36	81.68	32
FCN 16s	86.51	97.2	96.87	89.34	69.78	84.65	81.2	36
FCN 8s	86.95	97.17	97.08	89.17	70.63	85.13	82.52	36
Deeplab-largeFOV	86.36	97.17	96.94	89.28	68.19	84.51	82.08	13
Deeplabv2-VGG16	86.89	97.14	97.14	89.85	69.83	85.15	82.2	35
D-FCN 16s	86.47	97.2	97.02	89.71	68.23	84.75	81.9	30
D-FCN 8s	87.05	97.21	97.01	90.21	69.79	85.4	82.68	55
D-FCN 4s	87.11	97.22	97.16	89.92	70.51	85.05	82.78	173
Unet	86.81	97.17	96.98	89.67	69.95	84.55	82.51	20

**Figure 4:**
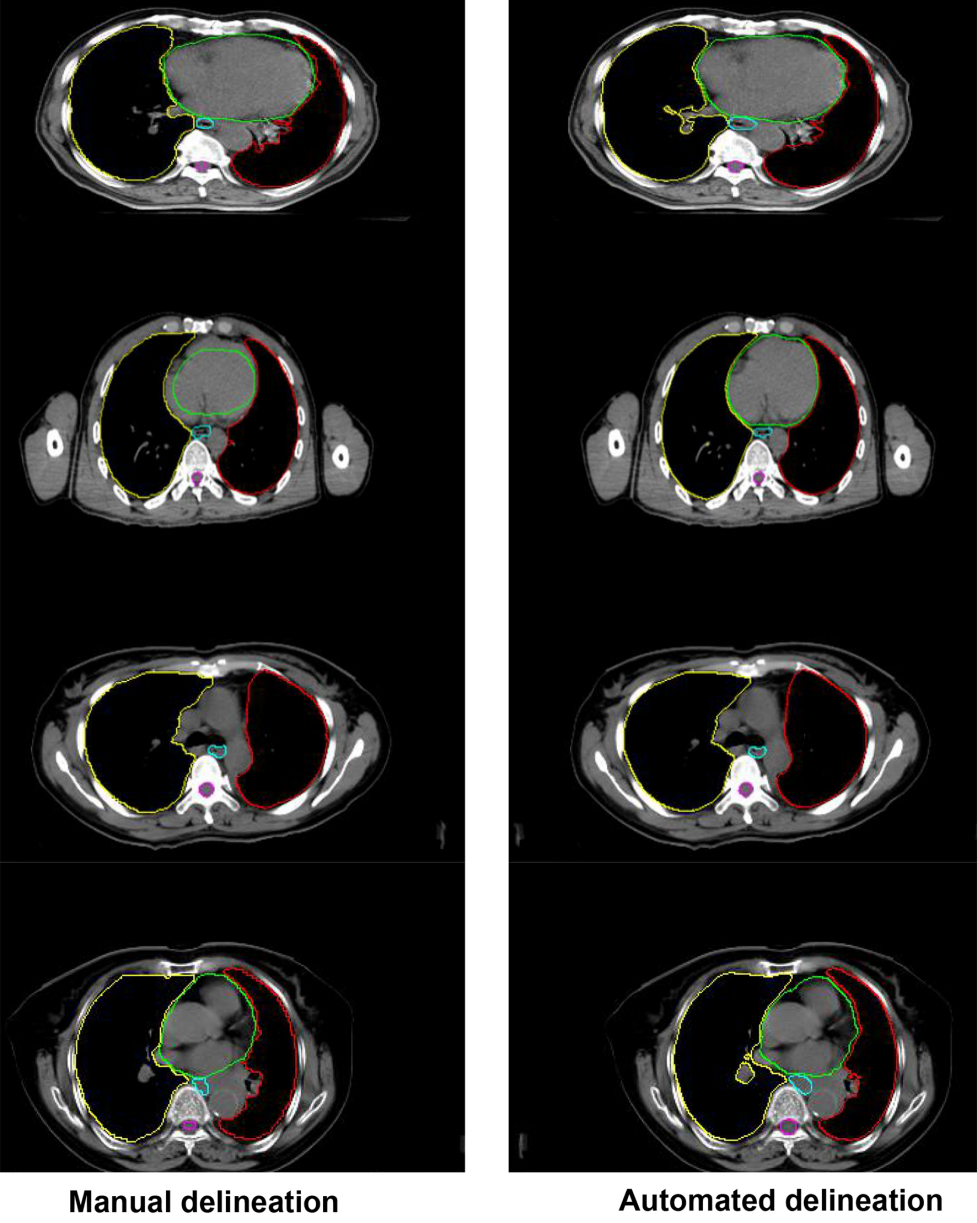
The comparison between the automated segmentation delineation of some test cases and the manual delineation results of the radiologist. In the figure, each horizontal line lists a comparison of different test cases. The left side is delineated by physicians and the right side by the D-FCN4s model automatically.

**Figure 4** shows the comparison between the results of automated segmentation delineation of some test cases and manual delineation by radiologists. In this figure, each horizontal line lists a comparison of different test cases. The left-side images were delineated by physicians and the right-side images by the D-FCN4s model automatically. The delineated contours of the both sides are very consistent with each other, especially for some closed esophageal or tracheal contours that are not easy to be distinguished by naked eyes. The trained D-FCN4s show good ability of predictive segmentation.

## Discussion

When designing a clinical radiotherapy plan, radiologists are required not only to accurately determine and delineate the tumor target area to be treated, but also to delineate the normal tissues and organs at risk that may be potentially irradiated. The accuracy of contouring organs at risk determines the quality of dose optimization in radiotherapy planning (15), thus directly affecting the success of radiotherapy or the incidence of complications (16). However, the accuracy of manual delineation is highly dependent on the clinical experience of radiologists, whose manual work might be inefficient (17–19). Therefore, automated organ delineation methods based on image segmentation have been attracting the tremendous interest of many scholars, who developed many different automated image segmentation and delineation algorithm models. Nonetheless, so far, most of the automated partition and delineation software commonly used in radiotherapy clinically are regional segmentation methods based on regional features such as gray level distribution (20), and the template automated delineation method based on empirical atlas and deformation model (21). The former is not effective for regional segmentation for little variation in gray level distribution, while the latter is sensitive to template quality, and the delineation effect is not good enough to meet the clinical requirements. Relevant studies (22) indicated that the mean Dice values of template-based automated partition delineation software using single and multiple template delineation, in the automated delineation of the geometric accuracy of organs at risk in head and neck radiotherapy, were 0.68 ± 0.20 and 0.74 ± 0.16, respectively (P = 0.01). Peng Yinglin et al. (23) found in the pre-clinical test report of the same automated delineation software that when the image to be delineated is significantly different from the template image, the Dice score of automated organ delineation was only 0.46 ~ 0.89. In recent years, the emerging deep learning method based on neural networks can automatically learn features and perform feature recognition at multiple levels, which has achieved great results in the application of automated recognition and segmentation of medical images. Ilsang Woo et al. (24) used a convolutional network method for automated segmentation of magnetic resonance images, and the Dice/% was greater than 85%, significantly higher than that of the traditional algorithm. Khalifa F et al. (25) employed a multiscale convolutional network method to perform automated segmentation of the kidneys on abdominal CT images with a Dice/% of 97.27%. In this paper, the pre-training and tuning methods of deep learning were leveraged to pre-train and optimize the published DCNN models such as FCN and DeepLab and the Unet model suitable for natural image segmentation, which were further improved by optimizing the FCN network model and combining the atrous convolution method. After the models were fully tuned and trained, the automated segmentation test was performed with another set of image in the same category. The results showed that the performance of the improved network model in automated organ segmentation was better than that of the other published models. The D-FCN 4S model proposed in this study brought about results that were very close to those of manual delineation in most segmentation of organ structures. The test experiment showed that in terms of individual organ structure, this D-FCN 4S model had the highest accuracy in automated segmentation of lung and pericardium, with an Dice of 97% and 89%, respectively. The similarity between automated and manual delineation of trachea and esophagus was relatively low, with an Dice of 70.51% and 85.05%, respectively. Since there is often great disagreement when physicians delineate organs such as the esophagus in the closed state (19, 26–29), using these datasets with great disagreement to train network models might potentially reduce the automated recognition ability of the models. At the same time, individual differences in physician delineation could somewhat reduce the consistency of automated delineation tests (30, 31). Therefore, when using machine learning tools like artificial intelligence automated delineation, it is necessary to label and optimize the data for deep learning models, and the results from automated delineation still need to be confirmed and modified by physicians. In addition, the results of this study revealed that the ability of the model to recognize and segment some small organ structures is relatively poor, and thus we need more efforts for debugging of parameters and iteration deepening when training and optimizing the models. We should seek more appropriate network parameters and iteration endpoints to improve the automated recognition ability and segmentation accuracy of the model. Besides, the cross-validation with smaller bias should be performed in the future studies. These are the issues that need to be addressed in our subsequent studies.

## Conclusion

This study introduced DCNN based on natural image segmentation into medical image segmentation and proposed an modified D-FCN that could effectively improve the ability of predictive segmentation of target images. Combined with GPU hardware acceleration, further optimization of network parameters and training levels might be expected to achieve rapid segmentation of images of organs at risk in the thoracic radiotherapy planning, thus paving the ground for automated design of radiotherapy plans in the future.

## Data Availability Statement

The original contributions presented in the study are included in the article/**Supplementary Material**. Further inquiries can be directed to the corresponding author.

## Author Contributions

HX and QL: substantial contributions to the conception and design of the work. HX, J-FZ, and QL: acquisition, analysis, and interpretation of data for the work. HX: drafting the work. QL, revising it critically for important intellectual content. HX, J-FZ, and QL, agreement to be accountable for all aspects of the work in ensuring that questions related to the accuracy or integrity of any part of the work are appropriately investigated and resolved. All authors contributed to the article and approved the submitted version.

## Funding

This study was supported by: Science and Technology Funding Project of Chenzhou City, Hunan Province China (No. ZDYF2020165) (QL); Science and Technology Funding Project of Hunan Education Department, China (No.1 8C1023) (HX); Science and Technology Funding Project of Hunan Province, China (No. 2020SK52201) (HX); Key Laboratory of Tumor Precision Medicine, Hunan colleges and Universities Project (2019-379) (QL).

## Conflict of Interest

The authors declare that the research was conducted in the absence of any commercial or financial relationships that could be construed as a potential conflict of interest.

## Publisher’s Note

All claims expressed in this article are solely those of the authors and do not necessarily represent those of their affiliated organizations, or those of the publisher, the editors and the reviewers. Any product that may be evaluated in this article, or claim that may be made by its manufacturer, is not guaranteed or endorsed by the publisher.

## Abbreviations

FCN, fully convolutional network; AC, atrous convolution; D-FCN, dilation fully convolutional networks; DCNN, Deep convolutional neural network; CNN, convolutional neural network; GPU, graphic processing units; CT, computed tomography.
